# HigA2 (Rv2021c) Is a Transcriptional Regulator with Multiple Regulatory Targets in *Mycobacterium tuberculosis*

**DOI:** 10.3390/microorganisms12061244

**Published:** 2024-06-20

**Authors:** Mingyan Xu, Meikun Liu, Tong Liu, Xuemei Pan, Qi Ren, Tiesheng Han, Lixia Gou

**Affiliations:** 1 Hebei Province Key Laboratory of Occupational Health and Safety for Coal Industry, School of Public Health, North China University of Science and Technology, Tangshan 063210, China; xumingyan0518@163.com (M.X.); liumeikun2019@126.com (M.L.); liutt_1206@163.com (T.L.); 18861999241@163.com (X.P.); renqi@ncst.edu.cn (Q.R.); 2 School of Life Science, North China University of Science and Technology, Tangshan 063210, China

**Keywords:** toxin–antitoxin, *Mycobacterium tuberculosis*, transcriptional regulation, persistence

## Abstract

Toxin-antitoxin (TA) systems are the major mechanism for persister formation in *Mycobacterium tuberculosis* (*Mtb*). Previous studies found that HigBA2 (Rv2022c-Rv2021c), a predicted type II TA system of *Mtb*, could be activated for transcription in response to multiple stresses such as anti-tuberculosis drugs, nutrient starvation, endure hypoxia, acidic pH, etc. In this study, we determined the binding site of HigA2 (Rv2021c), which is located in the coding region of the upstream gene *higB2* (*Rv2022c*), and the conserved recognition motif of HigA2 was characterized via oligonucleotide mutation. Eight binding sites of HigA2 were further found in the *Mtb* genome according to the conserved motif. RT-PCR showed that HigA2 can regulate the transcription level of all eight of these genes and three adjacent downstream genes. DNA pull-down experiments showed that twelve functional regulators sense external regulatory signals and may regulate the transcription of the HigBA2 system. Of these, Rv0903c, Rv0744c, Rv0474, Rv3124, Rv2603c, and Rv3583c may be involved in the regulation of external stress signals. In general, we identified the downstream target genes and possible upstream regulatory genes of HigA2, which paved the way for the illustration of the persistence establishment mechanism in *Mtb*.

## 1. Introduction

At present, tuberculosis (TB) remains a global infectious disease that is difficult to fully cure and is the second leading cause of death from a single pathogen [1]. TB is caused by *Mtb* infection. *Mtb* possesses a survival mechanism known as persistence, which means it can enter a persist state under external stress conditions such as antibiotics, with basic metabolic activities ceasing, rendering antimicrobial drugs ineffective [2]. After persistence is generated, most bacteria are sensitive to antibiotics, and only a very small part survive. After re-inoculation culture, most of the persistent bacteria were sensitive to antibiotics, and only a very small part survived [3]. Presently, almost all of the antibiotics used are aimed at growing bacteria and are basically ineffective for persisters. Since the formation of persistent bacteria does not require genetic mutation, but rather only the need to enter a “dormant” state, theoretically, all bacteria can be persistent, resulting in prolonged illness and easier relapse. At the same time, the persistent bacteria have long-term resistance to the environment of antibacterial drugs and are prone to gene mutation and drug resistance [3,4].

The bacterial TA system is a key factor in inducing the formation of persisters [5]. There are at least 80 TA systems in *Mtb*, and type II is the main type [6]. Type II TA systems are selectively degraded by host proteases such as Lon and ClpCP under stress conditions, allowing the toxin proteins to act. Antitoxin proteins of type II systems such as MqsRA, RelBE, HigBA, and other families are transcriptional regulators [7,8].

It has been found that certain antitoxins can regulate the transcription of several genes outside their own TA systems, including other TA systems [9]. The TA system is therefore likely to have transcriptional regulators. Thus, there may be transcriptional regulatory networks of TA systems that can generate complex stress responses to multiple external stresses. Among the many TA systems, HigBA2 has long been predicted as a pair of TA systems [10], but studies have not identified a toxic phenotype for the toxin HigB2 [11] and protein interactions between HigB2 and HigA2 have not been reported. One of the antitoxin genes, *higA2*, was predicted to be a transcriptional regulator, and ChIP-seq analysis showed that it may regulate the transcription of up to 25 genes [12]. After stressing *Mtb* with anti-TB drugs such as isoniazid, rifampicin, streptomycin, and ciprofloxacin, the transcript levels of at least 10 TA systems were found to be up-regulated up to 3-fold [13]. Of these, the HigBA2 system was identified as the key site that was activated under all conditions tested, and it could be starved by starvation conditions in addition to responding to anti-TB drugs [14], sustained hypoxia [15], and acidic environments [16]. Other TB foci and macrophage-like environments activate transcription, and all of these conditions are known to induce the formation of persisters. Therefore, the HigBA2 system may play a key role in the formation of *Mtb* persisters. In addition, the protein crystal structure of HigA2 has been resolved and was found to have N-terminal autocleavage activity that spontaneously removes the N-terminal’s 30 amino acids [17]. Although the three-dimensional protein structure of HigA2 has been characterized, its DNA recognition site has not been identified. In this study, we report the TA activity of HigBA2 in *Mtb* and the binding site of HigA2 to find its upstream and downstream regulatory genes.

## 2. Materials and Methods

### 2.1. Bacterial Strains, Plasmids, and Growth Conditions

*E. coli* Top10, the BL21(DE3) pLysS strain, and the *Mycobacterium smegmatis* (*M. smegmatis*) mc^2^155 strain were kept in the laboratory. The pJV53 plasmid and the pRSFDuet-1-*higA2* plasmid were purchased from GenScript (Nanjing, China). *E. coli* BL21(DE3) pLysS was used for protein expression and *E. coli* Top10 was used for gene cloning. Growth and virulence experiments were performed with *M. smegmatis* mc^2^155 as described previously [18]. *M. smegmatis* mc^2^155 was grown at 37 °C in 7H9 supplemented with 0.2% glycerol, 0.05% Tween-80, and 10% Middlebrook OADC. Expression was induced using 0.2% acetamide when grown in a liquid medium or on a 7H10 solid medium containing 0.5% glycerol and 10% OADC. *E. coli* strains were grown on liquid LB or solid LB medium at 37 °C and expression was induced using 0.8 M Isopropyl β-D-1-Thiogalactopyranoside (IPTG). Middlebrook 7H9, 7H10, and OADC were from BD (New Jersey, CA, USA).

### 2.2. Construction of mc^2^155 Heterologous Expression Vector

*higA2*, *higB2*, and *higBA2* were cloned between the *Nde*I and *EcoR*I (New England Biolabs, Hitchin, UK) sites of the pJV vector to construct the *Mycobacterium* shuttle plasmid using the Seamless Cloning Kit (Beyotime, Shanghai, China). The Seamless Cloning PCR primers used to amplify the target genes are listed in Table S1. The plasmid was added to the pJV vector along with 200 μL of mc^2^155 receptor cells together in a 2 mm electroshock cup (parameters: 2.5 kV, 1000 Ω, 25 vf). The 7H9 medium was added immediately after electric shock by Harvard Apparatus BTX (Holliston, MA, USA). The medium was resuscitated at 37 °C and 160 rpm for 3–4 h, and then coated on a 7H10 plate containing 50 ng/μL kanamycin (Kan, Vernon, CA, USA).

### 2.3. HigB2-HigA2 Neutralization Assay

*M. smegmatis* containing either individual or combined inducible expression plasmids was cultured at 37 °C for 2 days, and the OD_600_ was adjusted to 0.2 by a 10-fold gradient dilution as described in previous studies [18,19]. Each drop of 2.5 μL bacterial suspension was placed on a 100 mL 7H10 solid plate with or without the 0.2% acetamide inducer and the appropriate amount of antibiotics. The plates were incubated for 2 days at 37 °C, and the growth phenotypes were observed.

### 2.4. Protein Purification

The sequence of the coding region of the HigA2 protein was cloned into the *E. coli* expression vector pRSF-Duet-1 between the *Nde*I and *Xho*I sites and transformed into *E. coli* BL21(DE3) pLysS for protein overexpression using the calcium chloride method. Cell growth, induction, and harvesting were performed as described previously, followed by sonication and elution [20]. Purified proteins were desalted by a His Trap desalting column containing buffer (20 mM Tris-HCl, 150 mM NaCl, pH 8.0) and measured using SDS-PAGE. Proteins were stored in a stock buffer containing 20% glycerol at −30 °C.

### 2.5. EMSA

The DNA-binding capacity of proteins was assessed using modified EMSA as previously described [21]. DNA substrates for EMSA were obtained by PCR from *Mtb* H37Rv genomic DNA, and oligo fragments and primer sequences are shown in Table S1. The purified HigA2 protein (1 μM) and DNA substrate were added to the EMSA reaction buffer (20 mM Tris-HCl, 150 mM NaCl, pH 8.0) (10 μL), and the reaction was performed at 37 °C for 5 min. Then, 2 μL of the 6 × DNA loading buffer was added, mixed well, and directly subjected to agarose gel electrophoresis or native-PAGE.

### 2.6. RT-PCR Assays

The cDNAs of H37Rv wild-type and Δ*higA2* knockout strains (Shanghai Gene-optimal Science & Technology, Shanghai, China) were used as templates, and primers are shown in Table S1. The *sigA* gene was selected as the internal reference, and a fragment of approximately 200 bp of possible downstream regulatory genes of HigA2 was subjected to RT-PCR.

### 2.7. DNA Pull-Down

*Mtb* H37Rv genomic DNA was used as a template to design a 5′ biotin-labeled probe for the *Rv2023c-higB2-higA2* core promoter region. The labeled probe was purified using a gel recovery kit to recover the probe, pre-mixed with 5 µg of biotin-labeled DNA and 500 µg of H37Rv total protein (Shanghai Gene-optimal Science & Technology, Shanghai, China) on the ice. Next, 100 µL of BeyoMag™ Streptavidin Magnetic Beads (Beyotime, Shanghai, China) was pretreated with cool PBS, and the mixture of DNA and protein was added to the magnetic beads. The beads were resuspended and incubated at 4 °C for 1 h. The beads were centrifuged at 5000× *g* for 30 s, the supernatant was removed, the precipitate was collected, and the beads were washed five times with cool PBS and centrifuged at 5000× *g* for 1 min. Then, as much of the supernatant as possible was removed, 200 µL of PBS was added to resuspend the magnetic beads, and the proteins were identified by LC-MS/MS (Beijing Bio-Tech Pack Technology Company, Beijing, China).

## 3. Results

### 3.1. HigA2 Exhibits Toxicity in M. smegmatis

**Figure 1:**
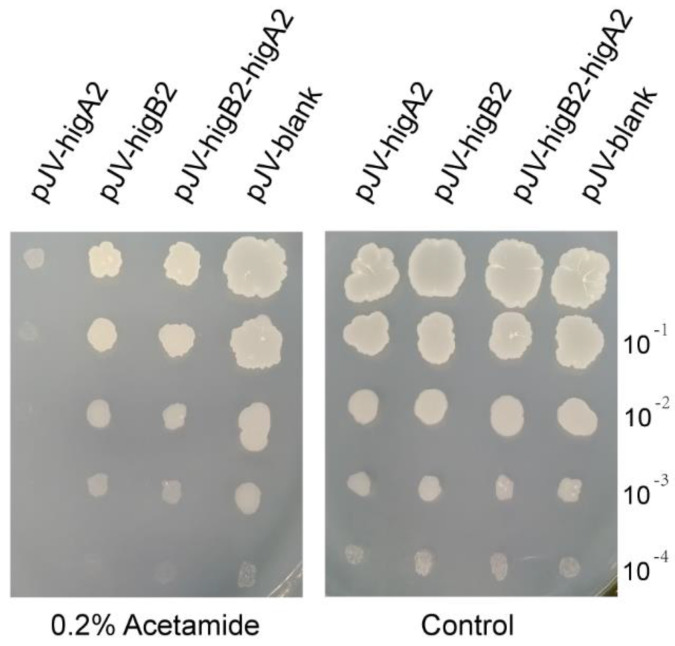
*M. smegmatis* growth performance check. Bacterial solution was adjusted to OD_600_ = 0.2. The resuspension was diluted 10^−1^, 10^−2^, 10^−3^, and 10^−4^-fold, and each diluted sample was spotted onto 7H10 solid medium with or without 0.2% Acetamide.

We cloned *higA2*, *higB2,* and *higB2-higA2* into pJV53 and successfully constructed the shuttle plasmid of *M. smegmatis* pJV-*higA2*, pJV-*higB2*, and pJV-*higB2*-*higA2*. The plasmid was transformed into *M. smegmatis* competent cells by electroporation. It was verified by colony PCR, and the primers are shown in Table S1. Acetamide was added to induce gene expression, and it was found that growth inhibition appeared in the *M. smegmatis* induced by the expression of *higA2* (Figure 1). HigB2 did not show obvious toxicity.

### 3.2. HigA2 Binding Site Identifications

**Figure 2:**
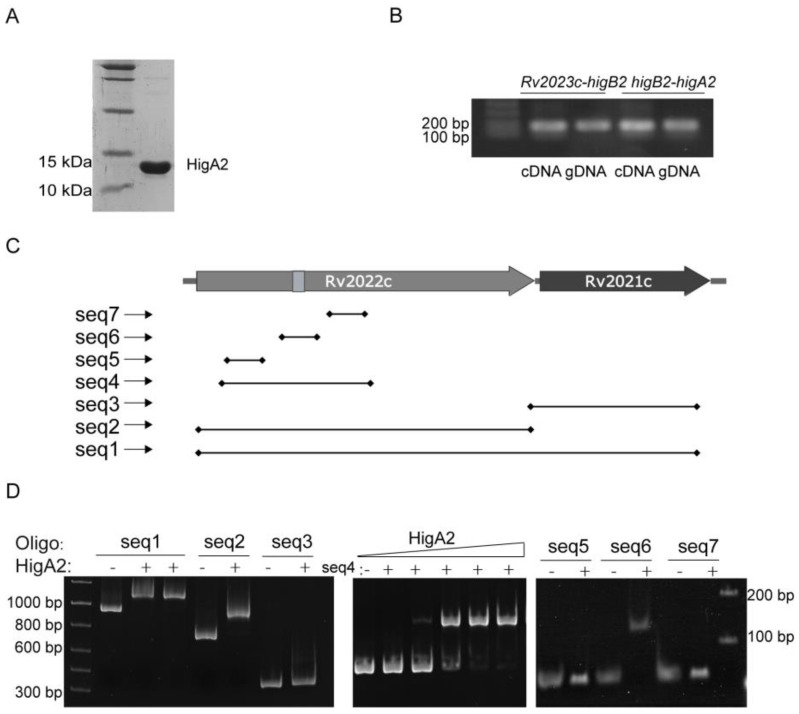
EMSA assay of HigA2 protein with possible binding sites. (**A**) C-terminal 6×His-tagged HigA2 was successfully expressed and purified. (**B**) PCR assay of *Rv2023c-higB2* and *higB2-higA2* intergenic regions in cDNA and gDNA of *Mtb* H37Rv. (**C**) The location of the HigA2 EMSA oligos. (**D**) EMSA detection of probe seq1-seq7 with HigA2.

HigA2 protein with a C-terminal 6×His-tag was successfully purified (Figure 2A). Reverse transcription PCR of the two-by-two spacer regions of *Rv2023c*, *higB2*, and *higA2* genes identified the existence of co-transcription of the three genes (Figure 2B). To determine the binding site of HigA2, the sequences 59 bp upstream of the coding frames of HigA2 and the DNA substrates *higA2*, *higB2*, and *Rv2023c* were subjected to an EMSA reaction, and the data did not show any binding of HigA2 to the three fragments. A DNA probe design was performed inside the *higB2-higA2* gene, the HigA2 protein and DNA probe were subjected to the EMSA reaction, and the DNA probe positions are shown here. Figure 2C shows that the HigA2 protein binds to the internal sequence seq2 of *higB2* (Figure 2D). The region was narrowed down to seq4. The three possible binding sites within *higB2*, namely seq5, seq6, and seq7, were further examined by EMSA, and the final binding sequence was determined to be seq6.

### 3.3. Identification of Conserved HigA2 Recognition Motifs

**Figure 3:**
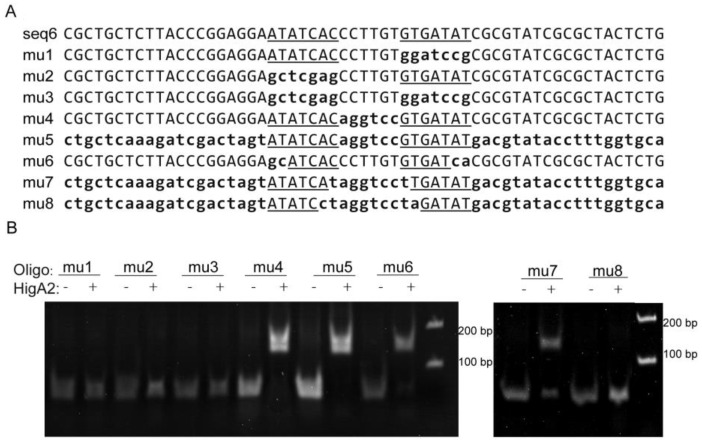
EMSA assay of HigA2 protein with promoter DNA mutants. (**A**) Sequences of seq6 and the eight mutants, with substitutions indicated by lowercase letters. Flag region is the inverted repeat sequence outlined and underlined, and the inter-region is the sequence between the two repeats. (**B**) EMSA experiments were performed on HigA2 and 8 mutant oligos.

The seq6 sequence has a palindromic motif formed by two inverted repeats (5′-ATATCAC(N)6GTGATAT-3′), and in order to confirm the importance of the specific recognition of this sequence, eight mutants originating from a DNA fragment of *higB2* promoter DNA with a length of 59 bp were used as substrates (Figure 3A). Among them, the flag region or inter-region of the m1 to m5 fragments were replaced by random sequences, respectively, or simultaneously, and the m6 fragment was reduced by two external bases. The two flag regions of the m7 fragment were reduced by one internal base, and the two flag regions of the m8 fragment were reduced by two internal bases. The results showed that HigA2 was able to bind to the DNA substrates mu4, mu5, mu6, and mu7 and weakly to DNA fragments with both binding site spacer lengths (8 bp), whereas neither a single binding site nor half of the palindromic sequence could bind to HigA2 (Figure 3B).

### 3.4. HigA2 Regulating Sites Exploration on the Genome Scale

**Figure 4:**
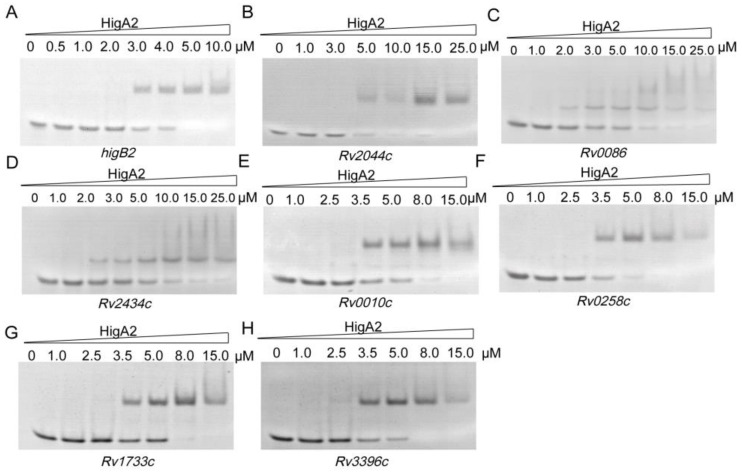
EMSA assay of HigA2 on the target oligos. (**A**–**H**) Eight oligos labeled with a length of 40 bp were all at a concentration of 1 μM and co-incubated with increasing concentrations of HigA2 protein.

Following the above results, we searched the *Mtb* H37Rv genome based on 5′-ATCAC(N)4GTGAT-3′, 5′-ATCAC(N)5GTGAT-3′, 5′-ATCAC(N)6GTGAT-3′, and 5′-ATCAC(N)7GTGAT-3′, and 31 possible binding sites were identified (Table S2). Thirty-one oligo fragments containing the above motifs were synthesized and subjected to an EMSA assay. Finally, it was determined that HigA2 could bind to eight oligos. Different genes were tested for their binding ability, and different concentrations of the HigA2 protein with regulatory motifs were subjected to EMSA experiments. HigA2 was able to bind essentially completely to *higB2*, *Rv2044c*, and *Rv0258c* at 5 μM; HigA2 was able to bind essentially completely to *Rv0010c*, *Rv1733c*, and *Rv3396c* at 8 μM. HigA2 binds essentially completely to *Rv0086* and *Rv2434c* at 20 μM (Figure 4).

The positions of the binding sites on the 11 genes and the functions of these genes are shown in Table 1, while the remaining genes may be involved in the establishment of the holding mechanism. Of these, Rv2043c is the proazinamidase *pncA*, which is responsible for the activation of the antibiotic proazinamide [22]. *Rv0086* and *Rv0087* are located within the *Rv0081*-*Rv0088* operon, which is involved in hypoxic adaptation [23,24]. Rv3396c is guanosine synthase *guaA*, an essential gene for basal metabolism, and is also involved in ppGpp signaling regulation [25]. Rv1733c is a membrane protein that has been found to act as a dormancy-associated surface antigen that activates the host immune system, with the potential to be developed as a vaccine for holdout bacteria [26]. Rv2433c is a secreted protein that can be recognized by T cells as an antigen [27]. Rv0010c is an unknown protein whose mutation is involved in pyrazinamide resistance [28].

**Figure 5:**
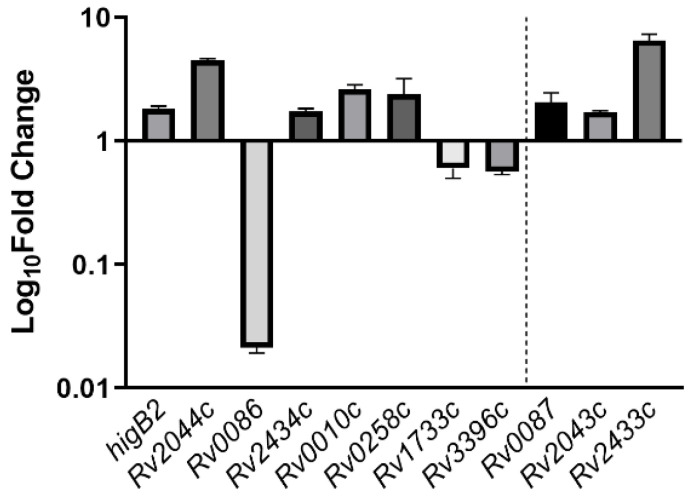
The expression of HigA2 downstream gene. The expression of HigA2 downstream genes was analyzed by reverse transcription PCR in the Δ*higA* mutant strain. The data are expressed as the relative fold expression of mRNA compared to *sigA*, the endogenous control.

The relative expression of all eight genes showed differences in the Δ*higA2* mutant strain. *higB2*, *Rv2044c*, *Rv2434c*, *Rv0010c*, and *Rv0258c* were up-regulated, while *Rv0086*, *Rv1733c*, and *Rv3396c* showed down-regulation. *Rv0086* showed significant down-regulation. For the binding site located at the end of the gene, *Rv0086* neighboring gene *Rv0087*, *Rv2434c* neighboring gene *Rv2433c*, and *Rv2044c* neighboring gene *Rv2043c* also appeared to be upregulated (Figure 5).

### 3.5. Exploration of Upstream Regulatory Genes of HigBA2

**Figure 6:**
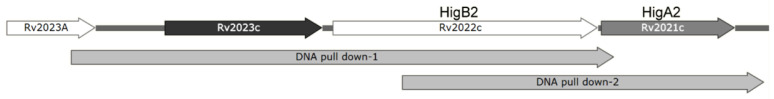
Illustration of DNA pull-down probes. Illustration of DNA pull-down-1 and DNA pull-down-2 in *Rv2023c-higB2-higB2*.

In order to find the gene that regulates HigA2 transcription, we designed two FAM fluorescence-labeled DNA probes for the 5′-UTR region of the HigA2 gene and performed a DNA pull-down assay on the total protein extract of *Mtb* H37Rv (Figure 6).

The samples obtained from the elution were subjected to protein profiling, and 412 proteins were identified that could bind to the above region. Among them, 12 were transcriptional regulators, and the specific information is shown in Table 2. All of the above-mentioned upstream regulatory genes may promote the formation of *Mtb*-holding bacteria by regulating the transcription of HigA2.

## 4. Discussion

Bacteria have evolved complex regulatory controls and multiple cellular transition states in response to a variety of environmental stresses. In order to survive, cells slow down their growth rate and redirect their metabolic resources until conditions are such that growth can be resumed [29,30]. The transcriptional activation mechanism of the TA system is essential for bacterial persistence, and although the mechanism of action can vary greatly and different DNA-binding domains and transcriptional regulatory mechanisms can be found even among members of the same TA family, toxin activity is diverse regardless of TA function and has been shown to interfere with basic cellular function [31].

### 4.1. HigBA2 Remains Uncleared for TA System Activity

Based on previous studies, the antitoxin HigA2 in *Mtb* acts as a transcriptional regulator with self-cleavage and structural flexibility and may bind DNA through HTH motifs [17]. Our study found that HigA2 binds within the *higB2* gene, and the expression of HigA2 in *M. smegmatis* resulted in growth inhibition of the bacteria, whereas the toxin HigB2 did not show toxicity.

Unfortunately, we did not successfully express HigB2 after replacing a variety of vectors, which may be due to inaccurate ORF prediction. Therefore, the binding activity of the antitoxin HigA2 and the toxin HigB2 is unknown. In *Mtb* and *E. coli*, the expression of the HigB1 toxin prevented bacterial growth and led to cell death [32,33,34]. After the induction of HigB1, a significant loss of viability was observed, leaving only a subset with potential acquisition persistence. tmRNA is a conserved target of HigB1 [32].

### 4.2. Downstream Regulatory Genes of HigA2 May Be Involved in the Establishment of Persistence

In the *Mtb* genome, HigA2 binds to and regulates a number of genes, six of which are bound to the interior of genes and five to the 5′-UTR region. TF regulates the transcription of genes in different ways by binding to CDS [35]. HigA2 regulates the transcription of these genes and may contribute to *Mtb* persistence.

*pncA* (*Rv2043c*) is co-transcribed into a polycistron along with *Rv2044c*, which is located 40 bp upstream of *pncA*. *pncA* encodes the pyrazinamidase enzyme, which is responsible for the conversion of an important first-line anti-TB drug, pyrazinamide (PZA), into its active form, and resistance to PZA is primarily due to mutations in *pncA* [36,37]. The detection of novel pyrazinamide-resistant mutations in clinical isolates of multidrug-resistant *Mtb* revealed novel non-synonymous mutations (Tyr70His, Ile71Asn) with effects on *PZase* activity in *Rv2044c*, all in the HigA2 binding site [38]. However, *Rv2044c* was significantly upregulated after *higA2* was knocked out and *pcnA* showed only slight upregulation. We hypothesized that *higA2* may influence *pcnA* to function under certain circumstances. The examination of intergenic distances suggests that the seven genes in *Rv0081-Rv0087* may form one operon [39]. *Rv0086* encodes proteins for the possible hydrogenase HycQ and is possibly involved in hydrogen metabolism. Based on the genetics of *M. smegmatis*, which induce the expression of their genes during starvation and hypoxia, they improve survival by scavenging atmospheric H_2_ [40,41]. *M. smegmatis* increased the transcription and synthesis of a form of dehydrogenase by 50-fold in response to organic carbon limitation, a process associated with persistence [42]. Guanosine monophosphate synthetase (GMPS), encoded by the *Rv3396c* (*guaA*), is a key enzyme in the biosynthesis of guanine nucleotides in *Mtb*. *guaA* is essential for the growth of *Mtb* H37Rv, and deletion of the *guaA* gene resulted in *Mtb* lethality [25]. In *E. coli*, *guaA* is essential for the formation of persister cells, as their absence significantly enhances cell sensitivity to various antibiotics [43]. GuaA acted as an upstream reaction in the ppGpp biosynthesis pathway and may affect persistence by disrupting ppGpp regulation [44].

Many anti-TB drugs, such as INH and EMB, target biological cell membranes to break down this barrier and weaken the bacteria. These anti-TB drugs that target the cell envelope can allow other drugs to pass through by interacting with the membrane. Importantly, many of the mutations that confer resistance to anti-TB drugs occur in these cell envelope biogenesis pathways, which is critical for new anti-TB drugs to combat TB resistance [45]. *Rv0010c* encodes a conserved membrane protein of unknown function that may play a role in the cell wall and cellular processes. There was a DnaA interaction site in the intergenic region of *Rv0010c-Rv0011c*, which was the same as the HigA2 binding site. At the same time, this study showed that the *mutation* of *Rv0010c-Rv0011c* in this position led to INH susceptibility. This mutation and *dnaA* mutation regulated the cell cycle and INH resistance in the same way [46]. *Rv2434c* may encode a conserved membrane protein, possibly cyclic glycine-binding proteins, involved in cAMP signaling pathway action [47]. The structure of Rv2433c (CFP11) has been studied [48]. It was identified as a human immunodominant T-cell antigen in a previous study [49], and CFP11 significantly increased immunoglobulin levels in human serum and promoted lymphocyte proliferation and interferon production [27]. Hypoxia-associated latency antigen Rv1733c is a possible integral membrane protein that stimulates cells to produce a certain level of cellular immunity, and this antigenic protein is readily recognized by the immune system of latent tuberculosis infection (LTBI) [50].

### 4.3. HigBA2 May Be Regulated by Multiple Upstream Proteins in Response to External Stresses

The results of DNA pull-down experiments suggest a possible response mechanism for *Mtb* during external stress. There are 12 transcription factors that may regulate the expression of HigBA2. These include two two-component signaling system regulators: Rv0903c (prrA) belonging to the prrAB two-component system in response to starvation conditions such as nitrogen source limitation [51], and Rv0744c, which responds to starvation conditions such as carbon source limitation [52]. Five induced variant transcriptional regulators, namely Rv3574 (TetR-family), Rv3833 (AraC-family), Rv2488c (LuxR-family), Rv3676 (Crp) sensed cAMP, and Rv1909c (FurA), could sense Fe^2+^. It was found that Rv2603c contributed to *Mtb* tolerance in macrophages [53]. Rv3124 (MoaR1) regulated molybdenum chitosan biosynthesis and was involved in hypoxic adaptation [54]. Rv0474 responded to Cu^2+^, inhibiting *rpoB* transcription to put the bacterium into dormancy [55]. Rv3583c (CarD) is a global transcriptional regulator that responds to starvation conditions [56]. These transcriptional regulators have the function of sensing external regulatory signals and may be involved in the transduction and regulation of external stress signals. However, their binding activities need to be further verified.

In summary, HigA2 regulates the expression of itself and multiple genes and may be involved in regulating *Mtb* hypoxia adaptation and the ppGpp pathway to promote persistence. Although the series of base substitutions on the DNA binding site in this study were able to illustrate the characteristics of the HigA2 recognition motif, more precise detection methods such as NMR titration or X-ray crystallography are still necessary to determine the accurate structure of the bound form of HigA2 and DNA, which is in the scope of our next research study. Due to lab limitations, we could not characterize the persistence difference between the wild H37Rv strain and the mutant Δ*higA2* strain since the cultivation of *Mtb* requires a BSL-3 laboratory. We will strive to seek cooperation with BSL-3 experimental platforms to conduct subsequent research such as persistence testing, co-cultivation with macrophages, etc. In our next study, the upstream regulatory genes of HigA2 should be further identified, and the regulatory mechanism of up-stream regulatory gene expression induced by external stress conditions such as anti-tuberculosis drugs, starvation conditions, and continuous hypoxia should be verified so as to determine the complete signal pathway and mechanism of *Mtb* persistence caused by external stress.

## 5. Conclusions

HigA2 may interfere with protein expression by binding to these genes, thereby affecting processes associated with *Mtb* persistence. There may exist a transcriptional regulatory network centered on *higA2* that regulates *Mtb*’s response to multiple external stresses and the transcription of multiple downstream genes, driving *Mtb* into a persistent state. Further study of this regulatory network can provide ideas for understanding the establishment of the *Mtb* persistence mechanism and the development of anti-TB drugs.

## Figures and Tables

**Table 1:** Conserved recognition motifs for HigA2.

Gene	Location	Feature	Illustration ^a^
*Rv2043c*	5′-UTR	*pncA*: Pyrazinamidase (PZase)	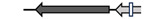
*Rv2044c*	CDS	relative with pyrazinamide resistance	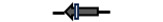
*Rv0086*	CDS	*hycQ*: hydrogenase HycQ	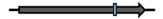
*Rv0087*	5′-UTR	*hycE*: Possible formate hydrogenase	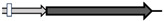
*Rv3396c*	CDS	*guaA*: GMP synthase	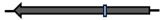
*Rv1733c*	CDS	transmembrane protein; surface antigen	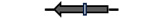
*Rv2433c*	5′-UTR	Secretory protein; T cell antigen	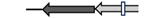
*Rv2434c*	CDS	transmembrane protein	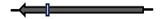
*Rv0010c*	5′-UTR	relative with pyrazinamide resistance	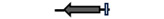
*Rv0258c*	5′-UTR	hypothetical protein	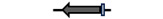
*higB2*	CDS	*higB2*: hypothetical protein	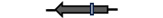

^a^ The dark arrow indicates the gene, the light arrow is the adjacent gene, and the white rectangle indicates the binding site.

**Table 2:** Transcription factors bound by *higBA2* pull-down.

Protern	Feature
Rv0903c	essential gene *prrA*, transcriptional regulator of the two component system PrrA/PrrB
Rv0744c	Possible transcriptional regulatory protein, similar to a two-component sensor
Rv3574	KstR, probable TetR-family transcriptional regulator involved in lipid metabolism
Rv3833	Probable AraC-family transcriptional regulatory protein
Rv2488c	Probable LuxR-family transcriptional regulatory protein
Rv3676	Crp, cAMP-activated global transcriptional regulator
Rv1909c	FurA, ferric uptake regulation protein, transcriptional regulator
Rv2166c	essential gene MraZ, transcriptional regulator
Rv3124	MoaR1, transcriptional regulator
Rv0474	Cu^2+^ responsive transcriptional regulator
Rv2603c	Probable transcriptional regulator
Rv3583c	essential gene CarD, RNA polymerase-binding transcription factor

## Data Availability

The original contributions presented in the study are included in the article and Supplementary Materials, further inquiries can be directed to the corresponding authors.

## Supplementary Materials

The following supporting information can be downloaded at: https://www.mdpi.com/article/10.3390/microorganisms12061244/s1, Table S1: Primers used in this study; Table S2: The distribution of target sites of HigA2 conserved recognition motifs in *Mtb* genome.

## References

[1] World Health Organization (WHO) (2023). Global Tuberculosis Report 2023.

[2] Boldrin F., Provvedi R., Cioetto Mazzabò L., Segafreddo G., Manganelli R. (2020). Tolerance and Persistence to Drugs: A Main Challenge in the Fight Against *Mycobacterium tuberculosis*. Front. Microbiol..

[3] Balaban N.Q. (2011). Persistence: Mechanisms for triggering and enhancing phenotypic variability. Curr. Opin. Genet. Dev..

[4] Zhang Y. (2014). Persisters, persistent infections and the Yin-Yang model. Emerg. Microbes Infect..

[5] Fasani R.A., Savageau M.A. (2013). Molecular mechanisms of multiple toxin–antitoxin systems are coordinated to govern the persister phenotype. Proc. Natl. Acad. Sci. USA.

[6] Tanaka M.M., Akarsu H., Bordes P., Mansour M., Bigot D.-J., Genevaux P., Falquet L. (2019). TASmania: A bacterial Toxin-Antitoxin Systems database. PLOS Comput. Biol..

[7] Ziemski M., Leodolter J., Taylor G., Kerschenmeyer A., Weber-Ban E. (2021). Genome-wide interaction screen for *Mycobacterium tuberculosis* ClpCP protease reveals toxin-antitoxin systems as a major substrate class. FEBS J..

[8] Kamruzzaman M., Wu A.Y., Iredell J.R. (2021). Biological Functions of Type II Toxin-Antitoxin Systems in Bacteria. Microorganisms.

[9] Soo V.W., Wood T.K. (2013). Antitoxin MqsA represses curli formation through the master biofilm regulator CsgD. Sci. Rep..

[10] Sala A., Bordes P., Genevaux P. (2014). Multiple toxin-antitoxin systems in *Mycobacterium tuberculosis*. Toxins.

[11] Mansour M., Giudice E., Xu X., Akarsu H., Bordes P., Guillet V., Bigot D.J., Slama N., D’Urso G., Chat S. (2022). Substrate recognition and cryo-EM structure of the ribosome-bound TAC toxin of *Mycobacterium tuberculosis*. Nat. Commun..

[12] Turkarslan S., Peterson E.J.R., Rustad T.R., Minch K.J., Reiss D.J., Morrison R., Ma S., Price N.D., Sherman D.R., Baliga N.S. (2015). A comprehensive map of genome-wide gene regulation in *Mycobacterium tuberculosis*. Sci. Data.

[13] Keren I., Minami S., Rubin E., Lewis K. (2011). Characterization and transcriptome analysis of *Mycobacterium tuberculosis* persisters. mBio.

[14] Betts J.C., Lukey P.T., Robb L.C., McAdam R.A., Duncan K. (2002). Evaluation of a nutrient starvation model of *Mycobacterium tuberculosis* persistence by gene and protein expression profiling. Mol. Microbiol..

[15] Bähler J., Rustad T.R., Harrell M.I., Liao R., Sherman D.R. (2008). The Enduring Hypoxic Response of *Mycobacterium tuberculosis*. PLoS ONE.

[16] Gupta A., Venkataraman B., Vasudevan M., Gopinath Bankar K. (2017). Co-expression network analysis of toxin-antitoxin loci in *Mycobacterium tuberculosis* reveals key modulators of cellular stress. Sci. Rep..

[17] Richardson W., Kang G.W., Lee H.J., Kwon K.M., Kim S., Kim H.J. (2021). Chasing the structural diversity of the transcription regulator *Mycobacterium tuberculosis* HigA2. IUCrJ.

[18] Chi X., Chang Y., Li M., Lin J., Liu Y., Li C., Tang S., Zhang J. (2018). Biochemical characterization of mt-PemIK, a novel toxin-antitoxin system in *Mycobacterium tuberculosis*. FEBS Lett..

[19] Agarwal S., Tiwari P., Deep A., Kidwai S., Gupta S., Thakur K.G., Singh R. (2018). System-Wide Analysis Unravels the Differential Regulation and In Vivo Essentiality of Virulence-Associated Proteins B and C Toxin-Antitoxin Systems of *Mycobacterium tuberculosis*. J. Infect. Dis..

[20] Gou L., Han T., Wang X., Ge J., Liu W., Hu F., Wang Z. (2017). A Novel TetR Family Transcriptional Regulator, CalR3, Negatively Controls Calcimycin Biosynthesis in Streptomyces chartreusis NRRL 3882. Front. Microbiol..

[21] Gao C.H., Yang M., He Z.G. (2011). An ArsR-like transcriptional factor recognizes a conserved sequence motif and positively regulates the expression of phoP in *Mycobacteria*. Biochem. Biophys. Res. Commun..

[22] Juréen P., Werngren J., Toro J.-C., Hoffner S. (2008). Pyrazinamide Resistance and pncA Gene Mutations in *Mycobacterium tuberculosis*. Antimicrob. Agents Chemother..

[23] Sun X., Zhang L., Jiang J., Ng M., Cui Z., Mai J., Ahn S.K., Liu J., Zhang J., Liu J. (2018). Transcription factors Rv0081 and Rv3334 connect the early and the enduring hypoxic response of *Mycobacterium tuberculosis*. Virulence.

[24] Kumar A., Phulera S., Rizvi A., Sonawane P.J., Panwar H.S., Banerjee S., Sahu A., Mande S.C. (2019). Structural basis of hypoxic gene regulation by the Rv0081 transcription factor of *Mycobacterium tuberculosis*. FEBS Lett..

[25] Villela A.D., Eichler P., Pinto A.F.M., Rodrigues-Junior V., Yates Iii J.R., Bizarro C.V., Basso L.A., Santos D.S. (2015). Gene replacement and quantitative mass spectrometry approaches validate guanosine monophosphate synthetase as essential for *Mycobacterium tuberculosis* growth. Biochem. Biophys. Rep..

[26] Zhang L., Ma H., Wan S., Zhang Y., Gao M., Liu X. (2022). *Mycobacterium tuberculosis* latency-associated antigen Rv1733c SLP improves the accuracy of differential diagnosis of active tuberculosis and latent tuberculosis infection. Chin. Med. J..

[27] Eweda G., Suzuki D., Nagata T., Tsujimura K., Koide Y. (2010). Identification of murine T-cell epitopes on low-molecular-mass secretory proteins (CFP11, CFP17, and TB18.5) of *Mycobacterium tuberculosis*. Vaccine.

[28] Shi W., Chen J., Zhang S., Zhang W., Zhang Y. (2018). Identification of Novel Mutations in LprG (rv1411c), rv0521, rv3630, rv0010c, ppsC, and cyp128 Associated with Pyrazinoic Acid/Pyrazinamide Resistance in *Mycobacterium tuberculosis*. Antimicrob. Agents Chemother..

[29] Moreno-Del Alamo M., Marchisone C., Alonso J.C. (2020). Antitoxin ε Reverses Toxin zeta-Facilitated Ampicillin Dormants. Toxins.

[30] Balaban N.Q., Helaine S., Lewis K., Ackermann M., Aldridge B., Andersson D.I., Brynildsen M.P., Bumann D., Camilli A., Collins J.J. (2019). Definitions and guidelines for research on antibiotic persistence. Nat. Rev. Microbiol..

[31] De Bruyn P., Girardin Y., Loris R. (2021). Prokaryote toxin-antitoxin modules: Complex regulation of an unclear function. Protein Sci..

[32] Schuessler D.L., Cortes T., Fivian-Hughes A.S., Lougheed K.E.A., Harvey E., Buxton R.S., Davis E.O., Young D.B. (2013). Induced ectopic expression of HigB toxin in *Mycobacterium tuberculosis* results in growth inhibition, reduced abundance of a subset of mRNAs and cleavage of tmRNA. Mol. Microbiol..

[33] Bordes P., Cirinesi A.-M., Ummels R., Sala A., Sakr S., Bitter W., Genevaux P. (2011). SecB-like chaperone controls a toxin–antitoxin stress-responsive system in *Mycobacterium tuberculosis*. Proc. Natl. Acad. Sci. USA.

[34] Gupta A. (2009). Killing activity and rescue function of genome-wide toxin-antitoxin loci of *Mycobacterium tuberculosis*. FEMS Microbiol. Lett..

[35] Hua C., Huang J., Wang T., Sun Y., Liu J., Huang L., Deng X., Chang Y.-F. (2022). Bacterial Transcription Factors Bind to Coding Regions and Regulate Internal Cryptic Promoters. mBio.

[36] Baddam R., Kumar N., Wieler L.H., Lankapalli A.K., Ahmed N., Peacock S.J., Semmler T. (2018). Analysis of mutations in pncA reveals non-overlapping patterns among various lineages of *Mycobacterium tuberculosis*. Sci. Rep..

[37] Mahmood N., Bhatti S., Abbas S.N., Shahid S., Nasir S.B. (2021). The pncA gene mutations of *Mycobacterium tuberculosis* in multidrug-resistant tuberculosis. Biotechnol. Appl. Biochem..

[38] Hameed H.M.A., Tan Y., Islam M.M., Lu Z., Chhotaray C., Wang S., Liu Z., Fang C., Tan S., Yew W.W. (2020). Detection of Novel Gene Mutations Associated with Pyrazinamide Resistance in Multidrug-Resistant *Mycobacterium tuberculosis* Clinical Isolates in Southern China. Infect. Drug Resist..

[39] Bacon J., James B.W., Wernisch L., Williams A., Morley K.A., Hatch G.J., Mangan J.A., Hinds J., Stoker N.G., Butcher P.D. (2004). The influence of reduced oxygen availability on pathogenicity and gene expression in *Mycobacterium tuberculosis*. Tuberculosis.

[40] Berney M., Greening C., Conrad R., Jacobs W.R., Cook G.M. (2014). An obligately aerobic soil bacterium activates fermentative hydrogen production to survive reductive stress during hypoxia. Proc. Natl. Acad. Sci. USA.

[41] Berney M., Cook G.M. (2010). Unique flexibility in energy metabolism allows *Mycobacteria* to combat starvation and hypoxia. PLoS ONE.

[42] Cordero P.R.F., Bayly K., Man Leung P., Huang C., Islam Z.F., Schittenhelm R.B., King G.M., Greening C. (2019). Atmospheric carbon monoxide oxidation is a widespread mechanism supporting microbial survival. ISME J..

[43] Mohiuddin S.G., Massahi A., Orman M.A. (2022). High-Throughput Screening of a Promoter Library Reveals New Persister Mechanisms in *Escherichia Coli*. Microbiol. Spectr..

[44] Hauryliuk V., Atkinson G.C., Murakami K.S., Tenson T., Gerdes K. (2015). Recent functional insights into the role of (p)ppGpp in bacterial physiology. Nat. Rev. Microbiol..

[45] Batt S.M., Burke C.E., Moorey A.R., Besra G.S. (2020). Antibiotics and resistance: The two-sided coin of the mycobacterial cell wall. Cell Surf..

[46] Hicks N.D., Giffen S.R., Culviner P.H., Chao M.C., Dulberger C.L., Liu Q., Stanley S., Brown J., Sixsmith J., Wolf I.D. (2020). Mutations in dnaA and a cryptic interaction site increase drug resistance in *Mycobacterium tuberculosis*. PLoS Pathog..

[47] Johnson R.M., McDonough K.A. (2018). Cyclic nucleotide signaling in *Mycobacterium tuberculosis*: An expanding repertoire. Pathog. Dis..

[48] Bu L., Brooks C.L. (2008). De novo prediction of the structures of *M. tuberculosis* membrane proteins. J. Am. Chem. Soc..

[49] Sable S.B., Kumar R., Kalra M., Verma I., Khuller G.K., Dobos K., Belisle J.T. (2005). Peripheral Blood and Pleural Fluid Mononuclear Cell Responses to Low-Molecular-Mass Secretory Polypeptides of *Mycobacterium tuberculosis* in Human Models of Immunity to Tuberculosis. Infect. Immun..

[50] Zhang W., Jiang H., Bai Y.L., Kang J., Xu Z.K., Wang L.M. (2014). Construction and Immunogenicity of the DNA Vaccine of *Mycobacterium Tuberculosis* Dormancy Antigen Rv1733c. Scand. J. Immunol..

[51] Haydel S.E., Malhotra V., Cornelison G.L., Clark-Curtiss J.E. (2012). The prrAB Two-Component System Is Essential for *Mycobacterium tuberculosis* Viability and Is Induced under Nitrogen-Limiting Conditions. J. Bacteriol..

[52] Subbian S., Gautam U.S., Mehra S., Kaushal D. (2015). In-Vivo Gene Signatures of *Mycobacterium tuberculosis* in C3HeB/FeJ Mice. PLoS ONE.

[53] Gao L.-Y., Groger R., Cox J.S., Beverley S.M., Lawson E.H., Brown E.J. (2003). Transposon Mutagenesis of Mycobacterium marinumIdentifies a Locus Linking Pigmentation and Intracellular Survival. Infect. Immun..

[54] Mendoza Lopez P., Golby P., Wooff E., Garcia J.N., Garcia Pelayo M.C., Conlon K., Gema Camacho A., Hewinson R.G., Polaina J., Suárez García A. (2010). Characterization of the transcriptional regulator Rv3124 of *Mycobacterium tuberculosis* identifies it as a positive regulator of molybdopterin biosynthesis and defines the functional consequences of a non-synonymous SNP in the Mycobacterium bovis BCG orthologue. Microbiology.

[55] Raghunandanan S., Ramachandran R., Gomez R.L., Devanarayanan S., Bommakanti A., Kondapi A.K., Varadarajan R., Kumar R.A. (2018). Rv0474 is a copper-responsive transcriptional regulator that negatively regulates expression of RNA polymerase β subunit in *Mycobacterium tuberculosis*. FEBS J..

[56] Li X., Chen F., Liu X., Xiao J., Andongma B.T., Tang Q., Cao X., Chou S.H., Galperin M.Y., He J. (2022). Clp protease and antisense RNA jointly regulate the global regulator CarD to mediate mycobacterial starvation response. eLife.

