# Supplementary material for: HigA2 (Rv2021c) Is a Transcriptional Regulator with Multiple Regulatory Targets in Mycobacterium tuberculosis
Source: Microorganisms. 2024 Jun 20;12(6):1244. doi: 10.3390/microorganisms12061244 (PMC11205783; doi:10.3390/microorganisms12061244)
Supplement: Supplementary file 1 [file microorganisms-12-01244-s001.zip › microorganisms-3051445-supplementary.pdf]

**Table S1 Primers used in this study**

| Primer | 5’ Sequence 3’ |
| --- | --- |
| seq1-F | GCGAAGGAGATATACATATGAACGTTCCATGGGAGAA |
| seq1-R | GGAATTCTCTAGAGATATCCTATGCCAGGGTGAATG |
| seq2-F | GCGAAGGAGATATACATATGAACGTTCCATGGGAGAA |
| seq2-R | GGAATTCTCTAGAGATATCTTACCCTTTCTTGCGCTTGCG |
| seq3-F | GCGAAGGAGATATACATATGGCGATGACACTACG |
| seq3-R | GGAATTCTCTAGAGATATCCTATGCCAGGGTGAATG |
| seq4-F | GGCACAGGCTACTGTTTCAAC |
| seq4-R | CGATAGCTGCGAACACCAA |
| seq5-F | CCTCGCCTGCCGGCATTTCCT |
| seq5-R | CCTCCGGGTAAGAGCAGCGGG |
| seq6-F | CGCTGCTCTTACCCGGAGGA |
| seq6-R | CAGAGTAGCGCGATACGCG |
| seq7-F | GACTCTGTGGGGTGGACGGC |
| seq7-R | TCCAAGCCGTTGAGCCAGGC |
| DNA pull down1-F | AAACGAGCAGGCTGGAACCG |
| DNA pull down1-R | GGCGTCCATGTCCCGTAGTG |
| DNA pull down2-F | TGCTCTTACCCGGAGGAAT |
| DNA pull down2-R | ATCTCGACCAACGGAGGG |
| *Rv2044c*-40bp-F | TGTCGCTCACTACATCACCGGCGTGATCTATCCCGCCGGT |
| *Rv2044c*-40bp-R | ACCGGCGGGATAGATCACGCCGGTGATGTAGTGAGCGACA |
| *Rv0086*-40bp-F | CGCCATCGCCGATATCACCGGCGTGATGCGACGGTCGCGG |
| *Rv0086*-40bp-R | CCGCGACCGTCGCATCACGCCGGTGATATCGGCGATGGCG |
| *Rv2434c*-40bp-F | GGTTCCCATGGGTATCACCTTCGTGATCGCCGGCAGCGTG |
| *Rv2434c*-40bp-R | CACGCTGCCGGCGATCACGAAGGTGATACCCATGGGAACC |
| *Rv0010c*-40bp-F | CAGTGGGGATGAATCACATGCGTGTGATTGAGTGACCAAA |
| *Rv0010c*-40bp-R | TTTGGTCACTCAATCACACGCATGTGATTCATCCCCACTG |
| *Rv0258c*-40bp-F | CATGGTGTCAGGATCACCCCGCTGTGATCAAAGATCAAGC |
| *Rv0258c*-40bp-R | GCTTGATCTTTGATCACAGCGGGGTGATCCTGACACCATG |
| *Rv1733c*-40bp-F | GTGTTGCTGTCGATCACCCCCTCGTGATCGATCACGGTCG |
| *Rv1733c*-40bp-R | CGACCGTGATCGATCACGAGGGGGTGATCGACAGCAACAC |
| *Rv3396c*-40bp-F | TGTGGGGTGTGCATCACCTCCGGGTGATACTGCACCCCGG |
| *Rv3396c*-40bp-R | CCGGGGTGCAGTATCACCCGGAGGTGATGCACACCCCACA |
| pJV-*higA2*-F | AGGGAGTCCACATATGGCGATGACACTACG |
| pJV-*higA2*-R | ACCGCGTCGGAATTCCTATGCCAGGGTGAATG |
| pJV-*higB2*-F | AGGGAGTCCACATATGAACGTTCCATGGGAGAA |
| pJV-*higB2*-R | ACCGCGTCGGAATTCTTACCCTTTCTTGCGCTTGCG |
| *higB2*-RT-F | GGCTTGGACCGAGAGACCTA |
| *higB2*- RT-R | GTCGAAGGCGAACAGAATGC |
| *Rv2044c*-RT-F | CCGCTCAGCTGGTCATGT |
| *Rv2044c*-RT-R | ACGTCCACCATACGTTCGGG |
| *Rv0086*-RT-F | CTGATCGTCCTGCTTGGCTT |

**Table S2 (continued)**

| | |
|---|---|
| *Rv0086*-RT-R | CAGCATGCGTCCGGAATTG |
| *Rv2434c*-RT-F | ACGTTTCTGGGCTTGACTGC |
| *Rv2434c*-RT-R | CCTTTGCCGCTCGTCGATAA |
| *Rv0010c*-RT-F | AGTCGTGATGGCTATTGCGT |
| *Rv0010c*-RT-R | TGTCGTAATAGCTGCGTCCG |
| *Rv0258c*-RT-F | GTAGCGAAGATGCTGCGGTT |
| *Rv0258c*-RT-R | GTTGAAGACCGGTCCGATGA |
| *Rv1733c*-RT-F | CGGTCAGCTGGTCGATGAAC |
| *Rv1733c*-RT-R | GAACAGGCTGTCGATGTCGT |
| *Rv3396c*-RT-F | ACCGGTTGACCTGTGTCTTC |
| *Rv3396c*-RT-R | GAACTGACGGCCGATGATCT |
| *Rv2043c*-RT-F | CAGAACGACTTCTGCGAGGG |
| *Rv2043c*-RT-R | GTGGCCACGACGAGGAATAG |
| *Rv0087*-RT-F | GAGCGTGGACTGATAGCGAC |
| *Rv0087*-RT-R | CCGGCACAACGTATTCCAAC |
| *Rv2433c*-RT-F | CCAAGCGATCGGTCTGTTTC |
| *Rv2433c*-RT-R | GATCACCATCCGTACCCACG |
| *sigA*-RT-F | TACGCTACGTGGTGGATTCG |
| *sigA*-RT-R | GTCTGGTCCAACGAGATCGG |

**Table S3 The distribution of target sites of HigA2 conserved recognition motifs in *Mtb* genome**

| Sequence | Location | Gene | Feature | Annotation |
|---|---|---|---|---|
| GCGATCACATCCGTGATCAC | 5'-UTR | Rv1908c | *katG* | catalase-peroxidase |
| CGCGATCACCGGCGTGATCACG | CDS | Rv3447c | *eccC4* | ESX-4 secretion system protein |
| GAGATCACCGAGGTGATCGC | CDS | Rv1133c | *metE* | methionine synthase |
| GATCACCACCGTGATC | CDS | Rv1237 | *sugB* | sugar ABC transporter permease |
| ATCACCACGGTGAT | CDS | Rv3061c | *fadE22* | acyl-CoA dehydrogenase FadE22 |
| TACATCACCGGCGTGATCTA | CDS | Rv2044c | | hypothetical protein |
| ATCACCGCCGTGAT | CDS | Rv2802c | | arginine/alanine-rich protein |
| ATCACCATCGTGAT | CDS | Rv2154c | *ftsW* | lipid II flippase FtsW |
| ATCACCATTGTGAT | CDS | Rv3063 | *cstA* | carbon starvation protein A |
| ATCACCGGCGTGAT | CDS | Rv3680 | | anion transporter ATPase |
| ATCACCGGCGTGAT | CDS | Rv0086 | *hycQ* | hydrogenase HycQ |
| ATCACCTTCGTGAT | CDS | Rv2434c | | transmembrane protein |
| AATCACATGCGTGTGATT | 5'-UTR | Rv0010c | | membrane protein |
| GATCACCCCGCTGTGATC | 5'-UTR | Rv0258c | | hypothetical protein |
| TCGATCACCCCCTCGTGATCGA | CDS | Rv1733 | | transmembrane protein |
| ATATCACCCTTGTGTGATAT | CDS | Rv2022c | *higB2* | hypothetical protein |
| GCATCACCTCCGGGTGATAC | CDS | Rv3396c | *guaA* | GMP synthase |
| CGTCATCACCCTGAGCGTGATGGCG | CDS | Rv3494c | *mce4F* | Mce family protein Mce4 |
| TGATCACCGGTGCGGTGATCA | CDS | Rv1850 | *ureC* | urease subunit alpha |
| GCGATCACGAGCATGGTGATCGC | CDS | Rv0507 | *mmpL2* | transmembrane transport protein |
| GCACGATCACGGTCCCGGTGATCGGGC | CDS | Rv0922 | | transposase |
| TCATCACCGACCTGGTGATGA | CDS | Rv0126 | *treS* | trehalose synthase/amylase TreS |
| GATCACCGGTTCGGTGATC | CDS | Rv2484c | | diacyglycerol O-acyltransferase |
| GATCACCCTTGAGGTGATC | CDS | Rv0568 | *cyp135B1* | cytochrome P450 Cyp135B1 |
| GATCACCGGGTGGGTGATC | CDS | Rv3061c | *fadE22* | acyl-CoA dehydrogenase FadE22 |
| GATCACCATGTGGGTGATC | | | | genomic position:2368742 |
| GATCACCGACGTCGTGATC | CDS | Rv1566c | | hypothetical protein |
| GATCACGGTGGTGGTGATC | IS | IS1607 | | insertion sequence |
| ATCACCGACGCGGTGAT | CDS | Rv1656 | *argF* | ornithine carbamoyltransferase |
| ATCACCACCGCGGTGAT | CDS | Rv1122 | *gnd2* | 6-phosphogluconate dehydrogenase |
| ATCACCCTGGTCGTGAT | CDS | Rv3005c | | hypothetical protein |
